# Physiologically variable ventilation reduces regional lung inflammation in a pediatric model of acute respiratory distress syndrome

**DOI:** 10.1186/s12931-020-01559-x

**Published:** 2020-10-31

**Authors:** Andre Dos Santos Rocha, Gergely H. Fodor, Miklos Kassai, Loic Degrugilliers, Sam Bayat, Ferenc Petak, Walid Habre

**Affiliations:** 1 Unit for Anaesthesiological Investigations, Department of Acute Medicine, University Hospitals of Geneva and University of Geneva, rue Willy Donzé 6, 1205 Geneva, Switzerland; 2 Department of Medical Physics and Informatics, University of Szeged, 9 Korányi fasor, Szeged, 6720 Hungary; 3 Department of Pediatric Intensive Care, Amiens University Hospital, Amiens, France; 4 Inserm UA7 STROBE Laboratory &, Department of Clinical Physiology, Sleep and Exercise, Grenoble University Hospital, Boulevard de La Chantourne, 38700 Grenoble, La Tronche France

**Keywords:** Mechanical ventilation, ARDS, Variable ventilation, Positron emission tomography, Regional ventilation

## Abstract

**Background:**

Benefits of variable mechanical ventilation based on the physiological breathing pattern have been observed both in healthy and injured lungs. These benefits have not been characterized in pediatric models and the effect of this ventilation mode on regional distribution of lung inflammation also remains controversial. Here, we compare structural, molecular and functional outcomes reflecting regional inflammation between PVV and conventional pressure-controlled ventilation (PCV) in a pediatric model of healthy lungs and acute respiratory distress syndrome (ARDS).

**Methods:**

New-Zealand White rabbit pups (n = 36, 670 ± 20 g [half-width 95% confidence interval]), with healthy lungs or after induction of ARDS, were randomized to five hours of mechanical ventilation with PCV or PVV. Regional lung aeration, inflammation and perfusion were assessed using x-ray computed tomography, positron-emission tomography and single-photon emission computed tomography, respectively. Ventilation parameters, blood gases and respiratory tissue elastance were recorded hourly.

**Results:**

Mechanical ventilation worsened respiratory elastance in healthy and ARDS animals ventilated with PCV (11 ± 8%, 6 ± 3%, p < 0.04), however, this trend was improved by PVV (1 ± 4%, − 6 ± 2%). Animals receiving PVV presented reduced inflammation as assessed by lung normalized [^18^F]fluorodeoxyglucose uptake in healthy (1.49 ± 0.62 standardized uptake value, SUV) and ARDS animals (1.86 ± 0.47 SUV) compared to PCV (2.33 ± 0.775 and 2.28 ± 0.3 SUV, respectively, p < 0.05), particularly in the well and poorly aerated lung zones. No benefit of PVV could be detected on regional blood perfusion or blood gas parameters.

**Conclusions:**

Variable ventilation based on a physiological respiratory pattern, compared to conventional pressure-controlled ventilation, reduced global and regional inflammation in both healthy and injured lungs of juvenile rabbits.

## Introduction

Acute respiratory distress syndrome (ARDS), characterized by the acute onset of severe hypoxic respiratory failure, remains a prevalent and often lethal condition in intensive care [1]. Although mechanical ventilation is a crucial life-saving treatment for ARDS, there is a considerable body of evidence indicating that prolonged positive-pressure ventilation can initiate, perpetuate or aggravate injury to lung tissue [2, 3]. The resulting exaggerated mechanical stress, along with the monotonous alveolar opening and closing, exerts shear stress and increased strain in the lung tissue [4], conditions that contribute to ventilator-induced lung injury (VILI).

While various modalities of mechanical ventilation have been proposed to reduce VILI [5–8], protective ventilation with monotonous tidal volume (VT) may not be the only rational strategy. In recent years, it has been advocated that mechanical ventilation reproducing the natural variability of breathing is better than conventional modes [9, 10]. Variable ventilation has been shown to be beneficial for gas exchange and respiratory mechanics in various animal models with healthy [11–13] or injured lungs, including ARDS [14–17]. We have previously established a variable ventilation modality using pre-recorded breathing patterns of healthy animals [18]. This physiologically variable ventilation (PVV) is characterized by breath-to-breath variability of VT and respiratory rate, in contrast to the monotonous conventional ventilation modes.

Recent interest in variable ventilation stems from the need to reduce cyclic alveolar reopening during mechanical ventilation, especially in injured lungs, to avoid development or propagation of lung inflammation, atelectasis and subsequent hypoxemia [19]. Whereas some studies demonstrated the beneficial effect of introducing variability into lung recruitment [20, 21], and others reported improvement in global respiratory mechanical and functional parameters [11–18], there is still a lack of detailed knowledge about the pathophysiological background related to the functional and regional behavior of the lung during variable ventilation. Moreover, the potential of PVV in the context of pediatric ARDS has not been characterized. To investigate the effect of PVV, lung functional and structural changes were compared to those obtained with conventional monotonous ventilation in normal lungs and ARDS, in a pediatric model. Global respiratory parameters were measured to characterize the overall lung condition. Regional lung aeration, pulmonary perfusion and inflammation were assessed by functional imaging using positron-emission tomography (PET) and single-photon emission computed tomography (SPECT) combined with X-ray computed tomography (CT).

## Methods

A more detailed description of the methods can be found in Additional file 1.

### Experimental animals

New Zealand White rabbit pups of both sexes, aged 4 to 5 weeks, were included in the present study (mean weight: 630 g, 370–860 g). This age can be approximated to an equivalent human age of 6 to 8 months [22]. Rabbits underwent tracheostomy and continuous intravenous (iv) anesthesia using propofol (10 mg/kg/h), fentanyl (5 µg/kg/h), midazolam (0.2 mg/kg/h) and atracurium (0.6 mg/kg/h).

### Study protocol

**Fig. 1:**
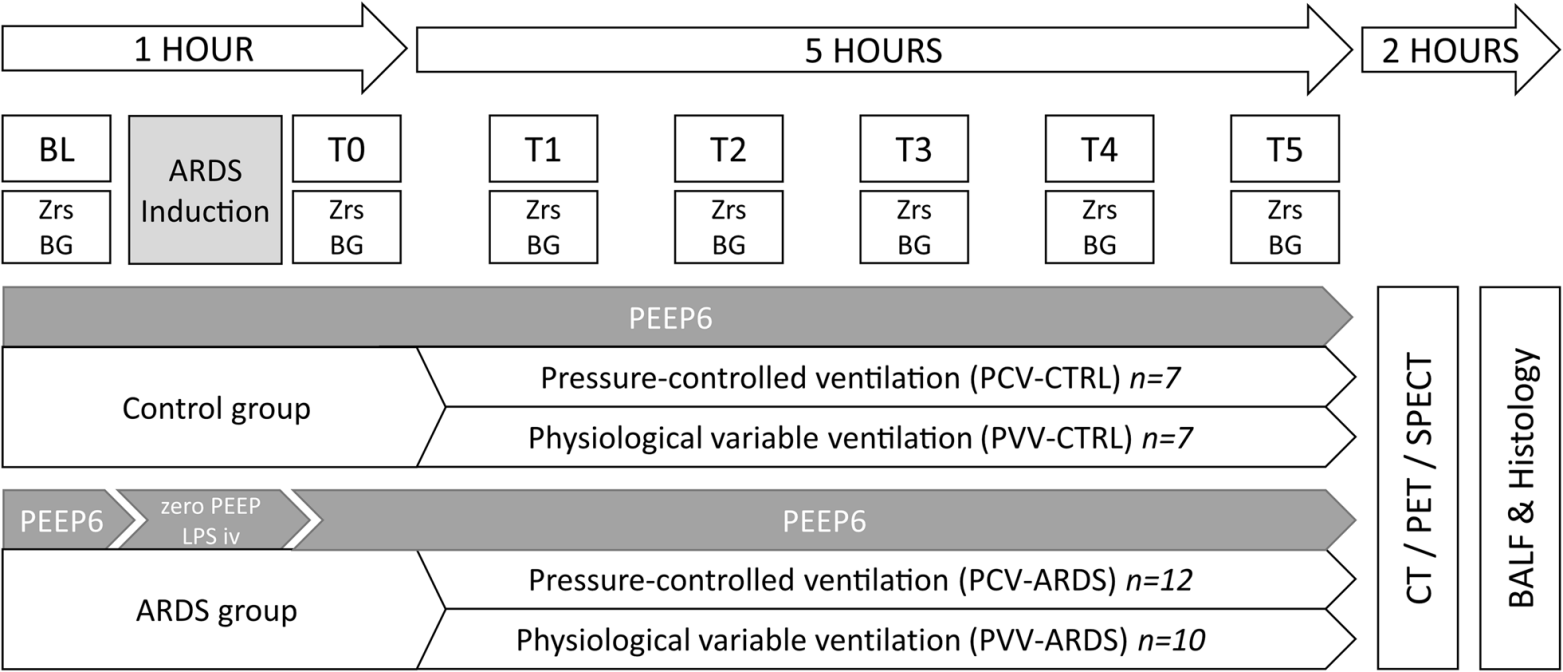
Schematic representation of the experimental protocol. BL: baseline; T0–T5: measurement timepoint at hour zero to hour five; Zrs: impedance of respiratory system; BG: blood gas; PEEP: positive end-expiratory pressure; LPS iv: lipopolysaccharide intravenous; PCV: pressure-controlled ventilation; PVV: physiological variable ventilation; CTRL: control; ARDS: acute respiratory distress syndrome

The protocol of the study is depicted in Fig. 1. Under baseline (BL) conditions, pressure-controlled ventilation was applied, using a positive end-expiratory pressure (PEEP) of 6 cmH_2_O, a fraction of inspired oxygen (FiO_2_) of 0.4, a VT of 8 ml/kg and a respiratory rate to achieve normocapnia (end-tidal CO_2_ of 5.5–6%). Arterial and central venous blood gas analyses and respiratory mechanical measurements were performed at BL. Subsequently, animals were randomized for the absence (CTRL) or presence (ARDS) of lung injury. Mild ARDS, according to the Berlin definition [23], was induced by combination of intravenous lipopolysaccharide (20 µg/kg) and injurious ventilation (VT = 40 ml/kg, 0 cmH_2_O PEEP, FiO_2_ = 1.0) with a target range of partial pressure of arterial oxygen (PaO_2_)/FiO_2_ ratio of 250–300 mmHg. When the target range of PaO_2_/FiO_2_ was reached, animals were further randomized for the ventilation mode: five-hour mechanical ventilation (VT = 8 ml/kg, PEEP = 6 cmH_2_O) was applied using either pressure-controlled ventilation (PCV) or PVV. FiO_2_ was adjusted according to PaO_2_/FiO_2_: using FiO_2_ = 0.4 above 250 mmHg; FiO_2_ = 0.6 between 200–250 mmHg; FiO_2_ = 0.8 between 100–200 mmHg, and FiO_2_ = 0.9 in the case that PaO_2_ decreased below 100 mmHg. Arterial blood gas and respiratory mechanics were measured hourly (T1–T5). After 5 h (T5), in vivo lung imaging was performed under continuous application of the ventilation mode. Subsequently, animals were euthanized with iv sodium thiopental (100 mg/kg). Bronchoalveolar lavage was performed ex vivo in the right lung, and the left lung was extracted for histological analysis.

### Experimental procedures

#### Measurement of respiratory mechanical parameters

Respiratory mechanical parameters were assessed by the wave-tube method of the forced oscillation technique, as detailed previously [14]. The constant-phase model [24] was fitted to the spectra to separate airway and tissue compartments of the respiratory system. Airway resistance (Raw), tissue damping (G) and tissue elastance (H) were estimated from the fits.

#### Application of physiologically variable ventilation

A commercially available pediatric ventilator (Servo-i, Maquet Critical Care, Solna, Sweden) was used with special firmware. The applied variable pattern was the reproduction of physiological breathing in rabbit pups, obtained using unconstrained whole-body plethysmography.

#### Lung imaging

Structural imaging of the respiratory system was acquired using CT. Regional lung perfusion was assessed though SPECT imaging using ^99m^Tc-labeled iv albumin macroaggregates. Regional distribution of inflammatory activity was assessed using PET imaging of fluorodeoxyglucose (^18^F-FDG) [25]. Lung radiodensity was expressed in mean pixel value (MPV), while PET and SPECT activity were expressed as standardized uptake value (SUV) normalized for voxelwise fraction of lung tissue [26].

CT images were segmented to well aerated, poorly aerated and non-aerated zones, based on radiodensity, as well as to ventral and dorsal halves. These segmented zones were considered when analyzing PET and SPECT images.

#### Measurements of secondary outcomes

Cell and cytokine content of the bronchoalveolar lavage fluid (BALF) was analyzed as detailed previously [18]. A histological lung injury score was determined according to the American Thoracic Society guidelines [27]. Tracheal pressure, airflow, arterial pressure, central venous pressure (CVP) and electrocardiogram were digitized and continuously recorded. Mean arterial pressure (MAP) and heart rate (HR) were assessed from these curves.

### Experimental outcomes

The primary outcomes of the present study were defined as respiratory mechanical parameters (Raw, tissue damping and elastance), arterial blood gas parameters (lactate, pH, PaO_2_/FiO_2_ and PaCO_2_) and imaging parameters. Secondary outcomes were hemodynamic and ventilation parameters, cytokine levels and lung injury histological indices.

### Statistical methods

Data are presented as mean ± half-width of 95% confidence interval. Normality of the data was assessed for each variable with the Shapiro–Wilk test. In case of a failed normality test, the variable was log-transformed. Repeated measures analyses of variance (ANOVA) using linear mixed-effect model fits by a restricted maximum likelihood (REML) method were applied to calculate statistical significances followed by Dunnett or Holm-Sidak post-hoc tests, using a significance level of p < 0.05, and all p values two-sided.

## Results

### Study population

Forty-four rabbits were randomized into one of four experimental groups. Eight rabbits were excluded from the analysis due to vital issues precluding the 5 h of ventilation (pneumothorax, n = 7; hemorrhage, n = 1). Therefore, 36 rabbits were included in the final analyses, with the following distribution: 12 rabbits were included in the PCV-ARDS group, 10 rabbits in PVV-ARDS, 7 rabbits in PCV-CTRL and 7 rabbits in PVV-CTRL.

### Respiratory mechanics

**Fig. 2:**
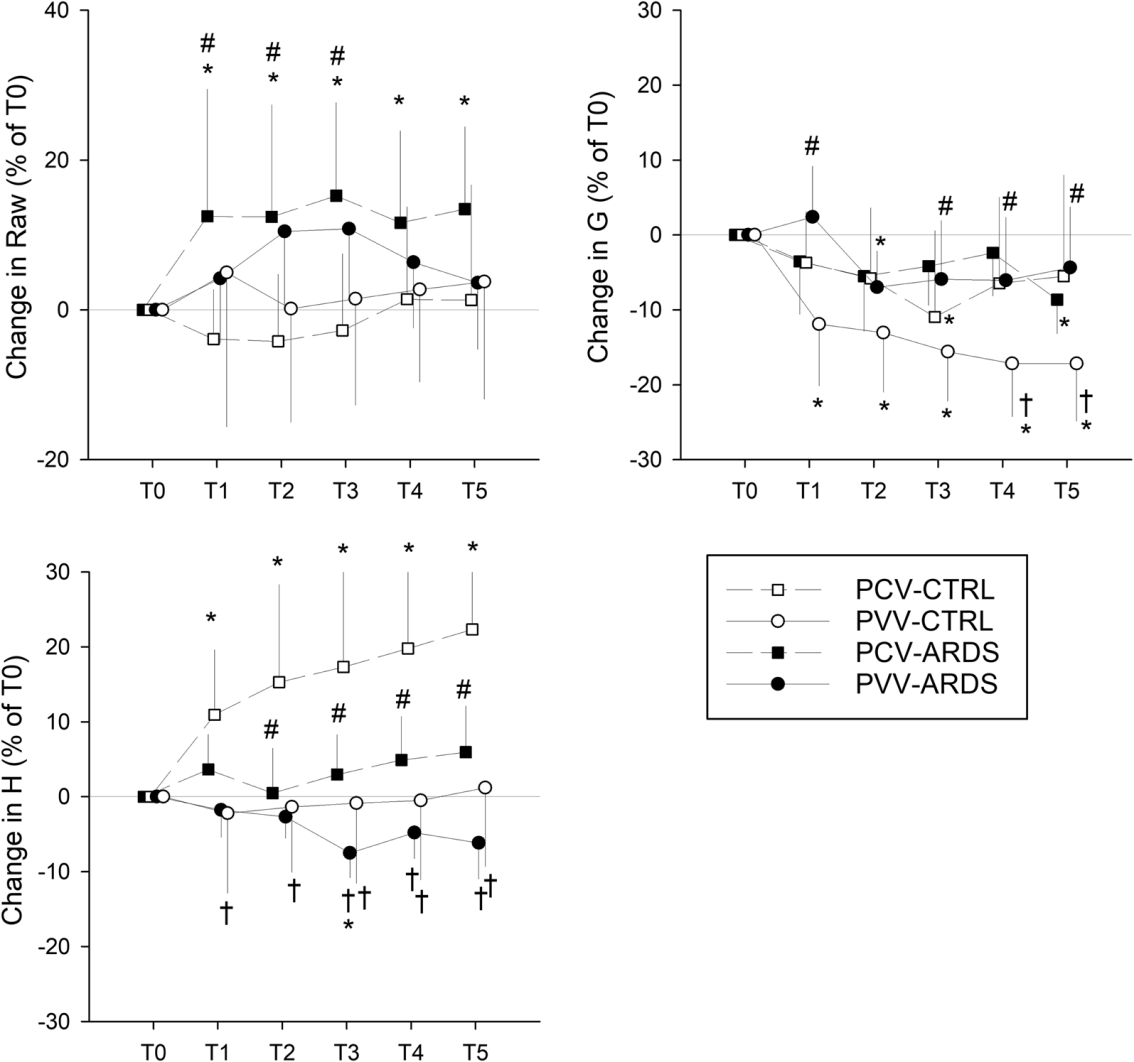
Changes in respiratory mechanical parameters relative to those obtained immediately after induction of lung injury (T0). Values expressed as mean ± half-width of 95% confidence interval. Raw: airway resistance; G: respiratory tissue damping; H: respiratory tissue elastance, T0: immediately after induction of lung injury; T1–T5: time points at the end of the corresponding hour of the 5-h long ventilation period; PCV: pressure-controlled ventilation; PVV: physiological variable ventilation; ARDS: presence of lung injury; CTRL: absence of lung injury. *p < 0.05 vs. T0, ^#^p < 0.05 vs. CTRL, ^†^p < 0.05 vs. PCV

Parameters characterizing respiratory mechanics obtained prior to initiating the 5-h ventilation are displayed in Additional file 1: Table S1. Changes in respiratory mechanical parameters relative to those obtained immediately after the induction of lung injury are displayed in Fig. 2. Applying PCV for 5 h led to significant increases in tissue elastance (T1–T5, p < 0.01) in the control animals and in Raw in the ARDS model (T1–T5, p < 0.03). Conversely, ventilating the lungs with PVV resulted in a significant decrease in tissue damping in control animals (T1–T5, p < 0.01), whereas no change in respiratory mechanics was detected in the ARDS model. Comparison of the two ventilation modes revealed significantly lower relative changes with PVV in tissue damping for the control animals (T4-T5, p < 0.03) and tissue elastance for the ARDS model (T1-T5, p < 0.01).

### Gas exchange

**Fig. 3:**
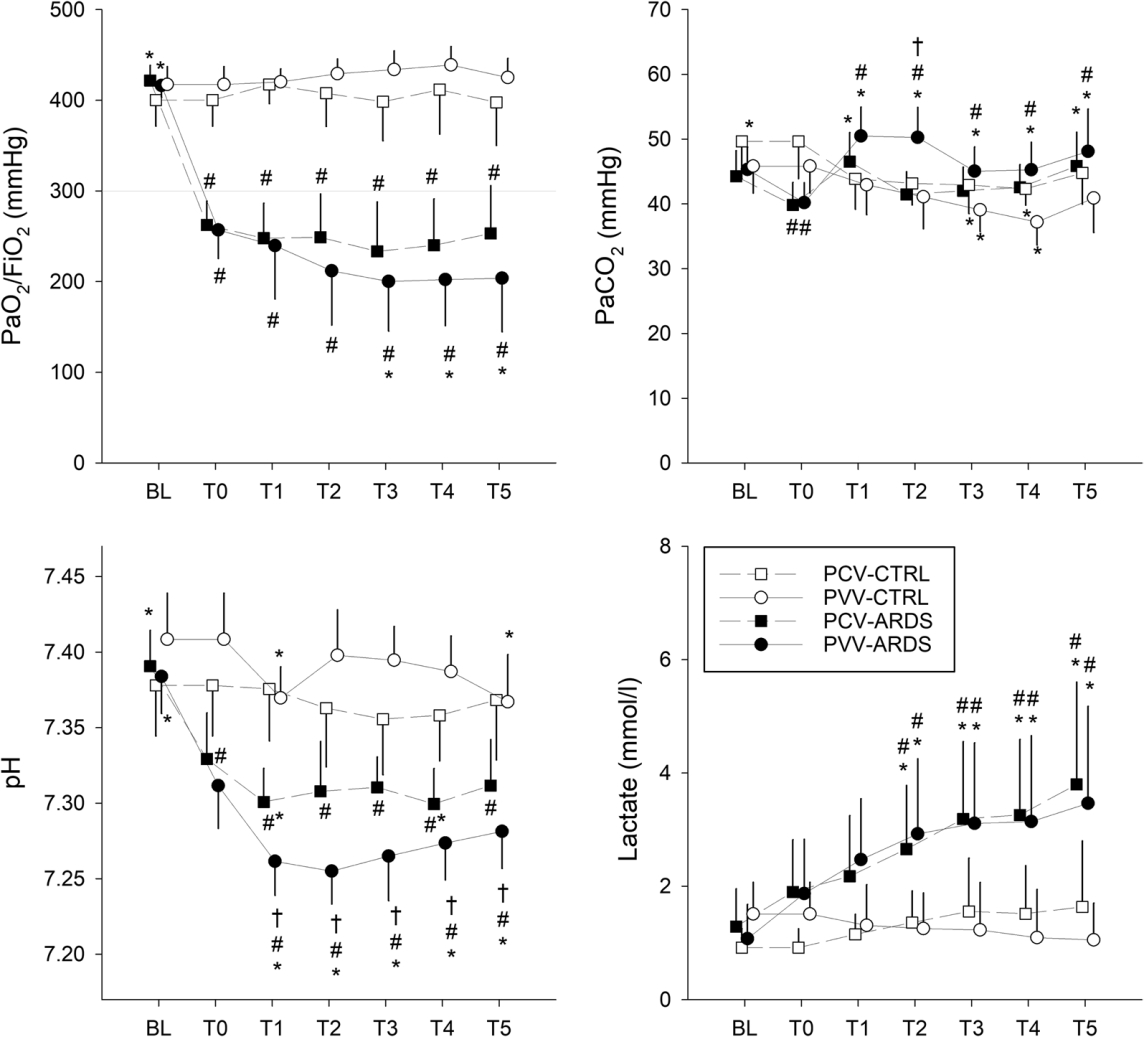
Blood gas parameters obtained before and during the 5-h ventilation period. Values expressed as mean ± half-width of 95% confidence interval. PaO_2_: partial pressure of arterial oxygen concentration; FiO_2_: fraction of inspired oxygen; PaCO_2_: partial pressure of arterial carbon dioxide concentration; BL: baseline; T0: immediately after induction of lung injury; T1–T5: time points at the end of the corresponding hour of the 5-h long ventilation period; PCV: pressure-controlled ventilation; PVV: physiological variable ventilation; ARDS: presence of lung injury; CTRL: absence of lung injury. *p < 0.05 vs. T0, ^#^p < 0.05 vs. CTRL, ^†^p < 0.05 vs. PCV

Figure 3 depicts the blood gas parameters during the 5-h ventilation. Inducing lung injury led to significant impairment of the blood oxygenation index (PaO_2_/FiO_2_), confirming the presence of mild to moderate ARDS, according to the Berlin definition [23]. Further drift in PaO_2_/FiO_2_ was observed in the PVV-ARDS group that resulted in statistically significant decreases after the third hour of mechanical ventilation (T3–T5, p < 0.045). Monotonous ventilation with PCV had no effect on the blood gas parameters in the control animals, whereas a systematic decrease in pH and plasma lactate concentration was observed in the ARDS groups (T1–T5, p < 0.001). Applying variable ventilation for 5 h in the control group had no systematic effect on gas exchange, whereas higher PaCO_2_ levels (T1–T5, p < 0.05) were associated with significantly diminished pH and elevated lactate in animals with ARDS (T1–T5, p < 0.01).

### Lung imaging

**Fig. 4:**
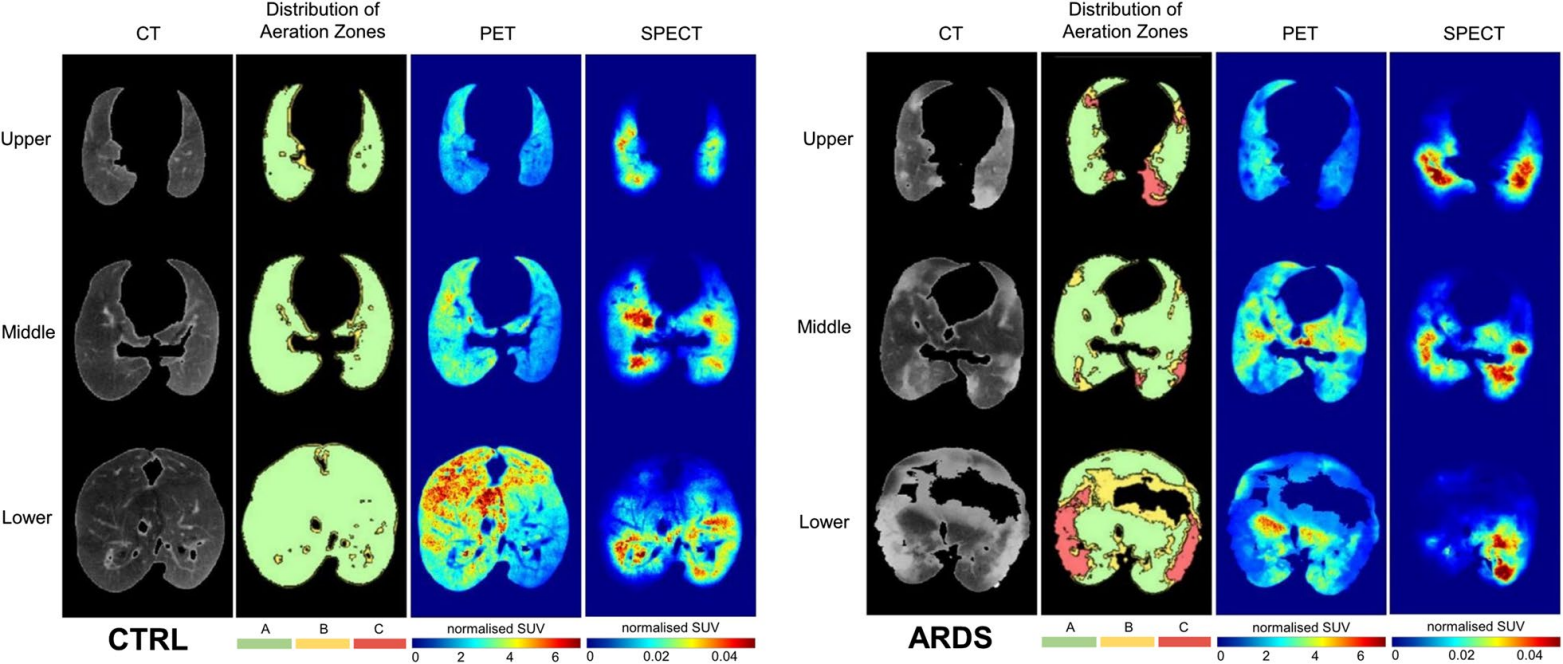
Representative images of CT, aeration maps, PET and SPECT (from left to right) in upper, middle and lower sections of the lung (from top to bottom) in the different experimental groups (CTRL and ARDS). Aeration zones A, B and C represent the well, poorly and non-aerated lung zones, respectively. PET and SPECT heating maps are represented in SUV normalized for tissue fraction for fludeoxyglucose and ^99m^Tc-labeled albumin macroaggregates, respectively

**Fig. 5:**
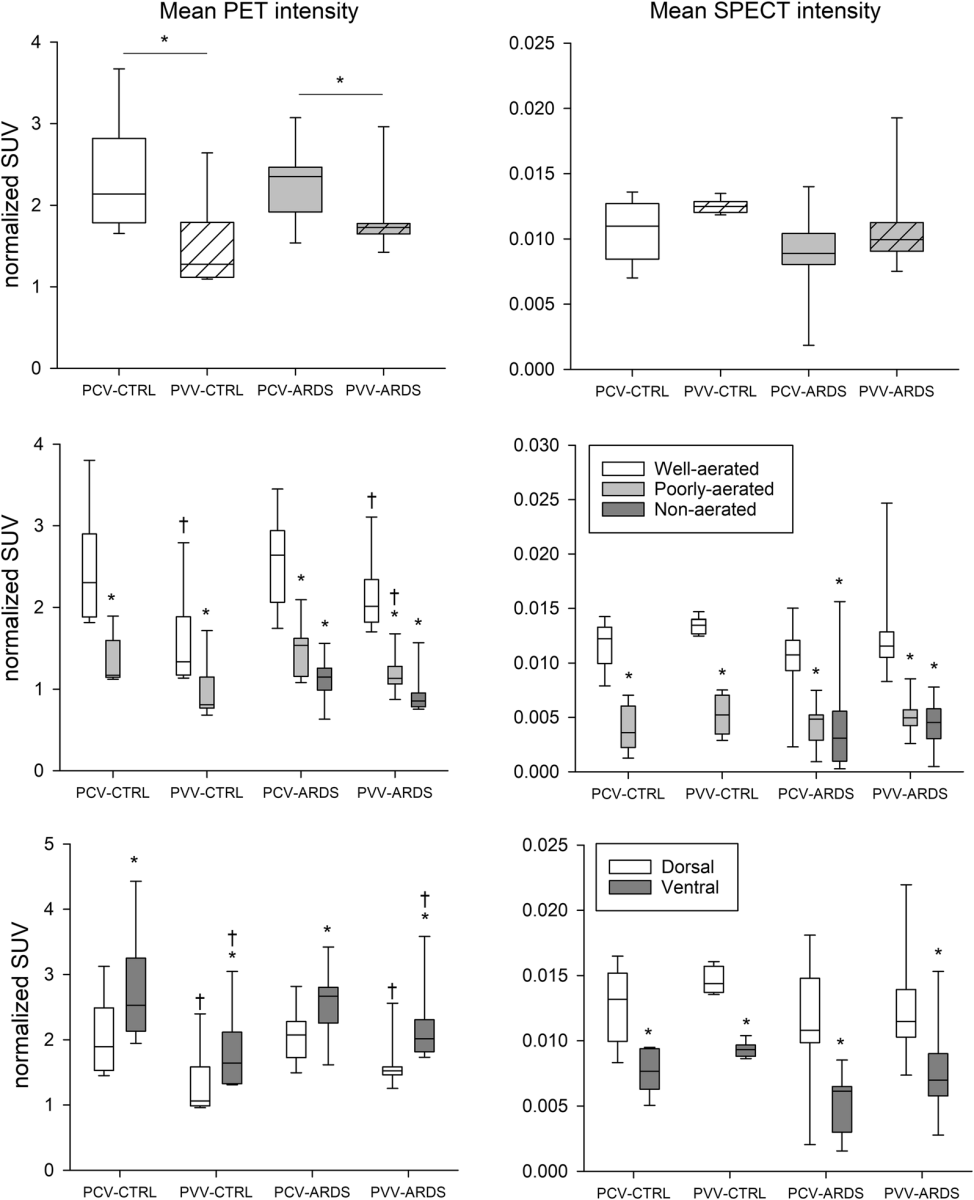
Left panels depict pulmonary inflammation characterized by PET imaging normalized to the tissue fraction. Right panels show pulmonary circulation characterized by SPECT imaging, normalized to tissue fraction. Upper panels represent mean PET and SPECT intensities averaged for the entire lung. Middle panels demonstrate the regional distribution based on aeration zones. Bottom panels represent the regional distribution based on the dependent (dorsal) and non-dependent (ventral) zones. SUV: standardized uptake value; PCV: pressure-controlled ventilation; PVV: physiological variable ventilation; ARDS: presence of lung injury; CTRL: absence of lung injury. *p < 0.05 vs. well-aerated or vs. dorsal, ^†^p < 0.05 vs. PCV

Representative CT, PET and SPECT images with the corresponding regional aeration maps in control and ARDS conditions are shown in Fig. 4. More heterogeneous lung structure, as indicated by heterogeneous regional distribution of ^18^F-FDG uptake and ^99m^Tc-labeled albumin macroaggregates, was observed in the presence of ARDS. The PET uptake values calculated for the total lung and at regional levels are summarized for the study groups in the left panels of Fig. 5. When averaging the entire lung, significantly lower mean ^18^F-FDG uptake was evidenced for the lungs in the animals ventilated with PVV, regardless of the presence of lung injury. This difference was also detected at the regional level in rabbits with healthy lungs ventilated with PVV (p < 0.04). Characterizing the differences in ^18^F-FDG uptake among the various aeration zones, defined by CT density, revealed the highest activity in the well aerated zones, with 2 to threefold differences compared to the non-aerated zones (p < 0.01, *well aerated vs. poorly aerated or non-aerated*). Likewise, ventral (non-dependent) regions presented significantly higher ^18^F-FDG uptake compared to dorsal (dependent) regions in both ventilation modes. Furthermore, significantly decreased mean ^18^F-FDG uptake was observed in the control animals ventilated with PVV compared to those with PCV (p < 0.01).

No evidence for a difference in SPECT activity was detected between the protocol groups (Fig. 5, right panels). However, regional perfusion was significantly and consistently higher in the well aerated zones and the dorsal zones of the lung, without differences between the experimental groups.

### Secondary outcomes

The detailed results on secondary outcomes (hemodynamic and ventilation parameters, cytokine levels and lung injury histological indices) can be found in Additional file 1. In the presence of ARDS, significantly higher driving pressure was required to maintain the same minute ventilation than in healthy animals (p < 0.01, ARDS vs. CTRL, Additional file 1: Figure S2). In the CTRL group, a progressive reduction in driving pressure was observed with PVV (p < 0.01 vs. T0, Additional file 1: Figure S2), which was not observed in animals ventilated with PCV.

No differences were detected between the two ventilation modes in regards of the hemodynamic parameters (Additional file 1: Figure S4), lung injury score (Additional file 1: Table S2), cytokine and cell content of BALF (Additional file 1: Table S3).

## Discussion

In the present study, a combined approach consisting of lung functional and structural assessment was used to investigate differences in the global and regional effects of PVV and the conventional monotonous pressure-controlled mode in a pediatric model of normal lungs and ARDS. The use of PVV decreased pulmonary inflammation, as assessed by ^18^F-FDG uptake, independent of lung condition. The decreased lung inflammation observed with PVV was also detected as an improvement in respiratory tissue elastance. Neither the use of PCV nor PVV affected blood gas and lung morphology indices.

Respiratory system mechanical parameters obtained in BL conditions or following induction of lung injury exhibited excellent agreement with previous data from the same species with similar weight range [14–16, 28]. Furthermore, the time course of the respiratory mechanical parameters over 5 h of ventilation in the control groups is in accordance with that observed previously in an experimental model using adult rabbits [18].

Since increases in tissue damping and elastance reflect lung volume loss and stiffening of the lung tissue [29, 30], the lack of an increase of elastance in the PVV-CTRL group suggests that lung derecruitment did not occur, and this conclusion is also supported by the lower inspiratory driving pressure achieved in this group. Moreover, the significant differences in elastance between the PCV-ARDS and PVV-ARDS groups observed after the 5-h ventilation suggest a protective effect of the variability on the conservation of lung volume in the presence of ARDS. Studies using models of mild-to-moderate lung injury have found similar beneficial effects on respiratory mechanics for variable ventilation [15, 31], and this protective effect was not observed in the presence of more severe ARDS [14].

Global and regional lung metabolic activity were measured by ^18^F-FDG uptake, a reliable biomarker of inflammation in the lung [32]. This marker is indicative of neutrophil activation in acute lung injury and ARDS [33–35]. Previous studies have shown that voxelwise ratio of lung parenchyma and air content influences ^18^F-FDG uptake quantification, requiring normalization for the tissue fraction [26, 36], which was performed in the current study. After 5 h of ventilation, we observed significantly lower indices of global and regional lung inflammation in the animals ventilated with PVV. Specifically, a significantly higher inflammatory activity characterized the well aerated and non-dependent lung zones, both in control and injured groups. This finding is consistent with results from previous experiments studying injured lungs, in which lung inflammation assessed by ^18^F-FDG uptake was correlated with regional strain [37, 38]. The significantly lower inflammation associated with PVV may be explained by the fact that the variability of the delivered VT contributes to tidal recruitment [12, 15], therefore reducing strain in the open, aerated zones. It is worth noting that PVV exerts the most beneficial effect in the well and poorly aerated zones under both control and ARDS conditions (Fig. 5). Conversely, the collapsed non-aerated zones were obviously unaffected by ventilation modes since these units were not subjected to strain. These findings further confirm the importance of focusing on regional ventilation when assessing the benefit of ventilation strategies. SPECT imaging confirms differences in regional distribution of lung perfusion when it is related to aeration zones. However, the lower blood perfusion in the ventral lung regions as compared to the dorsal zones can be attributed to the gravity effect and/or to the blood shift to the dorsal zones as a consequence of positive pressure and lung overdistension.

The beneficial effects of PVV on respiratory mechanics and lung inflammation were not reflected in changes in blood gas parameters. The lack of improvement in oxygenation may be related in part to the more severe hypoxemia in this group, which required a higher FiO_2_ (65% vs 55% in groups PVV-ARDS and PCV-ARDS, respectively). Moreover, the increase in lactate levels suggest the development of metabolic acidosis in both groups of ARDS animals, which may be the consequence of inadequate tissue oxygen delivery. Moreover, the timespan of the experiment (5 h) may be too short to detect effects on gas exchange. We may hypothesize that the more prominent inflammation observed in the PCV groups would build up and potentially cause gas exchange problems over the course of days.

The presence of ARDS was evident in the elevated lung injury score compared to control groups. In agreement with previous studies, lung injury score did not differ between the ventilation modes [14, 39]. The discrepancy between the functional and structural findings may be explained by the faster onset of functional changes, compared to the relatively longer time needed for morphological changes to become apparent. Lung inflammation quantified using BALF cell counts and pro-inflammatory cytokines, unlike in vivo imaging, did not reveal differences between the ventilation modes. In vivo imaging gives a more comprehensive measure of pulmonary inflammation at the early phase of ARDS, as it demonstrates the alveolar as well as the interstitial compartments of the lung. Additionally, ^18^F-FDG uptake reflects the acute metabolic activation of neutrophils and captures lung inflammation without barrier disruption, opposite to BALF neutrophils and cytokines, providing a more rapid assessment of inflammatory processes. In this context, it is worth noting that the control groups also showed increased inflammation and lung injury indices (BALF cytokines and histological injury score). These findings suggest that, despite the use of protective ventilation in the control groups, prolonged mechanical ventilation triggered the development of lung inflammation. This could potentially explain the lack of significant difference in normalized ^18^F-FDG uptake between control and ARDS lungs.

The similarity in the values of systemic hemodynamic parameters observed for the experimental groups is expected from the similarity in the overall lung perfusion as assessed by SPECT imaging. However, the significantly higher regional perfusion measured in the dependent zones can be attributed to the physiological distribution of lung perfusion that occurs in supine position [40] and is enhanced under positive pressure ventilation [41]. Considering the regional aeration of lung tissue, the significantly lower perfusion observed in the poorly and non-aerated zones can be explained by the hypoxic pulmonary vasoconstriction mechanism [42].

There are some methodological aspects of the present study that warrant consideration. In this study we used a Cone Beam CT [43]. This device uses less radiation and creates higher resolution images than the regular fan beam CT; however, it produces more scatter artefacts, which can alter the measured values [44, 45]. Due to technical limitations, breath gating was not performed in any of the acquisitions; therefore, basal lung areas had artefacts due to motion of the abdominal organs during breathing. The lung volume containing these artefacts was similar however, among rabbits.

The animal model to induce ARDS calls for some considerations as well. The components of the model were chosen to mimic the various pathophysiological aspects of ARDS observed in humans. Namely, intravenous LPS contributes to the inflammatory component of the disease and it has also been described to induce surfactant dysfunction [46]. Injurious ventilation using high VT combined with no PEEP contributes to development of volume- and barotrauma due to the supraphysiologic tidal volumes and respiratory pressures, whereas the absence of PEEP promotes tidal closures and exerts shear stress on the lung tissues [47]. The use of an FiO_2_ of 1.0 during this injurious ventilation period facilitates lung volume loss and development of ventilation heterogeneities [48]. While the surfactant dysfunction can restore to some extent during the 5-h timeframe of the experimental protocol, the functional and morphological damage is still present in the lungs, supported by the marked and highly significant changes observed between the control and ARDS groups regardless of the ventilation mode applied.

Measurements of respiratory mechanical parameters also warrant some considerations. While Raw is mainly specific to the flow resistance of the conducting airways [49], the tissue parameters damping and elastance include not only pulmonary components but are also influenced by other structures of the total respiratory system, mainly the chest wall [49]. Previous literature attributed a chest wall contribution of approximately 30–50% to these parameters [50] and since the chest wall contribution is not expected to change after lung injury and mechanical ventilation [51], the observed changes are interpreted as being mainly of pulmonary origin. Therefore, the corresponding changes registered in tissue damping and elastance are predictably underestimating the real pulmonary changes.

## Conclusions

Our data demonstrate the beneficial effect of variable ventilation based on a physiological breathing pattern in healthy lungs and in mild to moderate ARDS, in an experimental pediatric model. This positive effect was detected in the absence of deterioration in respiratory tissue elastance and in decreased regional lung inflammation measured by PET imaging. Ventilation for five hours with physiologically variable ventilation provided better protection on aerated lung zones than with monotonous pressure-controlled ventilation. While further studies in humans might be needed, our results suggest that the application of a physiological breathing pattern as the driving signal of mechanical ventilation may have a better lung protective ability than conventional modes in scenarios where prolonged mechanical ventilation is required.

## Supplementary information

**Additional file 1**. Supplementary information on methods and ancillary results (Figures S1–S4 and Tables S1–S3).

## Data Availability

The datasets used and/or analysed during the current study are available from the corresponding author on reasonable request.

## Abbreviations

ANOVA: Analyses of variance
ARDS: Acute respiratory distress syndrome
BALF: Bronchoalveolar lavage fluid
BL: Baseline
CT: X-ray computed tomography
CTRL: Control
CVP: Central venous pressure
FDG: Fluorodeoxyglucose
FiO_2_: Fraction of inspired oxygen
G: Tissue damping
HR: Heart rate
H: Tissue elastance
MAP: Mean arterial pressure
MPV: Mean pixel value
PaO_2_: Partial pressure of arterial oxygen concentration
PaO_2_/FiO_2_: Blood oxygenation index
PCV: Pressure-controlled ventilation
PEEP: Positive end-expiratory pressure
PET: Positron-emission tomography
PVV: Physiologically variable ventilation
Raw: Airway resistance
SPECT: Single-photon emission computed tomography
SUV: Standardized uptake value
T0–T5: Timepoint (hour) zero to timepoint (hour) five
VILI: Ventilator-induced lung injury
VT: Tidal volume
