# Supplementary material for: Physiologically variable ventilation reduces regional lung inflammation in a pediatric model of acute respiratory distress syndrome
Source: Respir Res. 2020 Oct 31;21:288. doi: 10.1186/s12931-020-01559-x (PMC7602830; doi:10.1186/s12931-020-01559-x)
Supplement: Supplementary file 1 — Additional file 1. Supplementary information on methods and ancillary results (Figures S1–S4 and Tables S1–S3). [file 12931_2020_1559_MOESM1_ESM.docx]

Additional file

Physiologically variable ventilation reduces regional lung inflammation in a pediatric model of acute respiratory distress syndrome

Andre Dos Santos Rocha^1*^, Gergely H. Fodor^1,2*^, Miklos Kassai^1^, Loic Degrugilliers^3^, Sam Bayat^4^, Ferenc Petak^2^, Walid Habre^1^

^1^ Unit for Anaesthesiological Investigations, Department of Acute Medicine, University Hospitals of Geneva and University of Geneva, rue Willy Donzé 6, 1205 Geneva, Switzerland

^2^ Department of Medical Physics and Informatics, University of Szeged, 9 Korányi fasor, 6720 Szeged, Hungary

^3^ Department of Pediatric Intensive Care, Amiens University Hospital, Amiens, France.

^4^ Inserm UA7 STROBE Laboratory & Department of Clinical Physiology, Sleep and Exercise, Grenoble University Hospital, Boulevard de la Chantourne, 38700 La Tronche, Grenoble, France

****: Equal contribution by the first two authors***

***Corresponding Author:***

Andre DOS SANTOS ROCHA, MD

Unit for Anaesthesiological Investigations

University of Geneva

6, rue Willy Donzé, 1205 Geneva

Switzerland

Ph: + 41 79 553 21 36

Fax: + 41 22 37 25 485

e-mail: Andre.DosSantosRocha@unige.ch

Number of figures: 5

**Keywords:** mechanical ventilation, ARDS, variable ventilation, positron emission tomography, regional ventilation

METHODS

**Ethical statement**

The experimental protocol was approved by the Experimental Ethics Committee of the University of Geneva and the Animal Welfare Committee of the Canton of Geneva, Switzerland (no. GE 64/17, modified by GE/144/17 and GE 30/18, 02 May 2017, 21 August 2017 and 19 February 2018). All procedures were performed according to the current animal protection laws of Switzerland (LPA, RS455) and reported in compliance with ARRIVE guidelines.

**Experimental animals**

New Zealand White rabbit pups of both sexes, aged 4 to 5 weeks, were included in the present study (mean weight: 630 g, 370–860 g). Animals were purchased from the farm of the University of Geneva (Arare, Geneva, Switzerland) and were delivered at least two days before the experiments to allow acclimatization. The rabbits had access to food and water *ad libitum* before the experiments and were kept in cages together with their mothers.

**Study design**

The protocol of the study is depicted in Figure 1 of the main manuscript. Following animal preparation, anaesthesia and surgery, ventilation was initiated in supine position using a commercially available neonatal ventilator (Servo‑i, Maquet Critical Care, Solna, Sweden) to apply a pressure-controlled mode, a positive end-expiratory pressure (PEEP) of 6 cmH_2_O, a fraction of inspired oxygen (FiO_2_) of 0.4, a VT of 8 ml/kg and a respiratory rate to achieve normocapnia (end-tidal CO_2_ of 5.5–6%). Before the beginning of the experimental protocol, two deep inflations (30 cmH_2_O peak pressure maintained for 5 seconds each) were applied to normalize lung volume history. Subsequently, following a 5-minute stabilization period, baseline (BL) measurements were carried out: arterial and central venous blood samples were obtained for blood gas analysis and respiratory mechanical properties were measured. Animals were randomized for the absence (CTRL) or presence (ARDS) of lung injury. Animals randomized to lung injury were exposed to a combination of intravenous injection of 20 µg/kg lipopolysaccharide (LPS, from *Escherichia coli* O111:B4, Sigma, Saint Louis, Missouri, USA) and injurious ventilation for 30 minutes (VT=40 ml/kg, PEEP=0 cmH_2_O, FiO_2_=1.0 with a respiratory rate to achieve the same minute ventilation as in BL) to provoke mild ARDS, according to the Berlin definition (1). Arterial blood gas analyses were performed to measure partial pressure of arterial oxygen (PaO_2_), and the results were used to assess the degree of injury. If the target range of PaO_2_/FiO_2_ ratio (250–300 mmHg) had not been achieved, injurious ventilation was repeated for further 10-minute periods. When the target range of PaO_2_/FiO_2_ was reached, collapsed alveoli were opened with 2 deep inflations (30 cmH_2_O peak pressure maintained for 5 seconds each), and, after a 5-minute stabilization period, another set of blood gas and respiratory mechanical data was collected (T0).

Animals were further randomized for the ventilation mode: five-hour mechanical ventilation was applied using either pressure-controlled ventilation (PCV) or PVV, at a PEEP of 6 cmH_2_O, an average VT of 8 ml/kg, and a minute ventilation to match that of BL. FiO_2_ was adjusted according to PaO_2_/FiO_2_: using FiO_2_=0.4 above 250 mmHg; FiO_2_=0.6 between 200–250 mmHg; FiO_2_=0.8 between 100–200 mmHg, and FiO_2_=0.9 in the case that PaO_2_ decreased below 100 mmHg. Arterial blood gas and respiratory mechanics were checked hourly (T1–T4) and ventilation was adjusted if necessary. After 5 hours (T5), a final set of arterial and venous blood gas samples and respiratory mechanical data was obtained. The animals were then transported to the imaging facility while undergoing sedation and mechanical ventilation. Lung imaging was performed under continuous application of the ventilation mode. When the imaging acquisition finished, animals were euthanized with a single injection of intravenous sodium thiopental (100 mg/kg). Bronchoalveolar lavage was performed *ex vivo* in the right lung, and the left lung was extracted for histological analysis.

**Experimental procedures**

All experiments were carried out at the laboratory of the Unit for Anaesthesiological Investigations, University Hospitals of Geneva and University of Geneva. Imaging procedures were carried out at the microPET/SPECT/CT Imaging Laboratory, at the Geneva site of the Centre d’Imagerie Biomédicale. Animal transport between the two research facilities was performed under sedation and mechanical ventilation. Because of time limitations of isotope production, animal preparation and mechanical ventilation were performed overnight to allow imaging acquisition during office hours.

*Anesthesia and surgical preparation*

Anesthesia was induced using intramuscular injection of xylazine (3 mg/kg) and ketamine (25 mg/kg) and maintained using a continuous intravenous infusion of propofol (10 mg/kg/h), fentanyl (5 µg/kg/h), and midazolam (0.2 mg/kg/h) through a 24 G catheter (Abbocath, Abbot Medical, Baar/Zug, Switzerland) in a marginal ear vein. Animals were placed on a thermostatic heating pad (Harvard Apparatus, South Natick, MA, USA) and a rectal thermometer probe was used to keep internal body temperature within the range of 38–39°C. Fluid balance of the animals was maintained by administering a 4 ml/kg/h continuous infusion of Ringer’s Acetate. Local anesthesia with lidocaine 1% was applied to the anterior cervical region, prior to a surgical tracheostomy using a 2.5-mm uncuffed tube (2.5 mm Portex, Smiths Medical, Kent, UK). The experimental procedures described below were performed under mechanical ventilation, using the pressure-controlled mode with a VT of 8 ml/kg, a respiratory rate to achieve normocapnia (5.5–6.0% end tidal CO_2_), a PEEP of 3 cmH_2_O and FiO2 of 0.4. A continuous infusion of atracurium (0.6 mg/kg/h) was initiated to induce neuromuscular blockade once proper depth of anesthesia was ensured.

The left femoral artery and right jugular vein were cannulated with a 20 G catheter (Abbocath, Abbot Medical, Baar/Zug, Switzerland) for invasive blood pressure measurements as well as, arterial and central venous blood gas sampling. Electrocardiogram was recorded using subcutaneous needle electrodes. Tracheal pressure, airflow, arterial pressure, central venous pressure (CVP) and ECG were digitized (sampling rate 1 kHz) and continuously recorded (ADInstruments, Powerlab model 8/35 and LabChart 7, Dunedin, New Zealand). Mean arterial pressure (MAP) and heart rate (HR) were calculated from the recorded traces.

*Measurement of respiratory mechanical parameters*

Respiratory mechanical parameters were assessed by the wave-tube method of the forced oscillation technique, as detailed previously (2). Briefly, a loudspeaker-in-a-box system was connected to the tracheal tube using a polyethylene wave-tube with known geometrical properties (length: 100 cm, internal diameter: 0.193 cm). A small amplitude (±1 cmH2O peak-to-peak pressure) forcing signal was generated by the loudspeaker and introduced to the respiratory system by short, 8-s long periods of apnea. The wave-tube and the chamber of the loudspeaker were pressurized to the level of PEEP to maintain the same mean airway pressure during the recordings. Two identical pressure transducers (Honeywell Differential Pressure Sensor model 24PCEFA6D, Charlotte, North Carolina, USA) were connected to the two ends of the wave-tube and lateral pressures were recorded using low-pass filtering at 25 Hz. Pressure signals were digitized at 256 Hz using an analog–digital data acquisition board (NI USB‑6211, National Instruments, Austin, TX, USA). Input impedance of the respiratory system (Zrs) was calculated from a fast Fourier transformation of the transfer function of the lateral pressures (3). Three to four comparable recordings were performed at each experimental step and the spectra obtained from these recordings were ensemble-averaged for further analysis.

Airway and tissue compartments of the respiratory system were separated by fitting the constant-phase model (4) to the spectra using a global optimization method. The model consists of an airway compartment with resistive (Raw, airway resistance) and inertive components (Iaw, airway inertance) in series with a constant-phase tissue compartment, including tissue damping (G) and tissue elastance (H). As established previously (5), Raw reflects the flow resistance of the central conductive airways, Iaw is related to the acceleration and deceleration of the air column in the central airways, tissue damping characterizes the energy loss within the respiratory tissues, and tissue elastance describes the energy storage properties of the respiratory tissues (elastance). The impedance of the instrumental dead space (including the measurement circuit and the tracheal tube) was measured and subtracted from the Zrs spectra before model fitting.

*Blood gas analyses*

Arterial and venous blood samples were analyzed by a point-of-care blood gas analyzer (i‑Stat, Abbott Laboratories, Chicago, IL, USA), and PaO_2_, PaCO_2_, pH and lactate concentrations were determined. Oxygenation index was calculated as PaO_2_/FiO_2_.

*Application of physiologically variable ventilation*

A commercially available pediatric ventilator (Servo‑i, Maquet Critical Care, Solna, Sweden) was used with special firmware. The ventilation pattern was applied via custom-made computer software in a looped manner. The applied variable pattern was the reproduction of physiological breathing in rabbit pups, obtained from a randomly selected rabbit pup using unconstrained whole-body plethysmograph. The rabbit was placed in a sealed transparent box that had an intentional leak to allow continuous supply of fresh gas. A pressure transducer was connected to the box (Honeywell Differential Pressure Sensor model 24PCEFA6D, Charlotte, North Carolina, USA) and the signal was digitized at 1 kHz (ADInstruments, Powerlab model 8/35 and LabChart 7, Dunedin, New Zealand) along with the feed of a digital camera pointed at the box. The internal temperature and humidity of the box were recorded. The rabbit was placed twice daily in the box for 40 min on five consecutive days to allow it to become accustomed to the box environment. Data recorded during the last 40-min period was used. Post-processing of the data included deletion data segments in which movement artefacts (verified by the recording of the camera feed) and sniffing behavior occurred. The resulting 10-min recording of spontaneous breathing was used as a driving pattern of the variable ventilation. Replicates with different average respiratory rates were created of a single pattern, by retaining the original ratios of breath-to-breath pressure and frequency. An inspiratory to expiratory (I:E) time ratio of 1:2 was used. The ventilation was adjusted to achieve an overall average VT of 8 ml/kg and an overall rate was selected to achieve normocapnia (5.5–6.0% end tidal CO_2_). Ventilation was checked and adjusted hourly, if necessary. The applied variable ventilatory pattern is presented in Figure S1, along with its characteristics throughout the study in Figures S2 and S3.

*Lung imaging*

Structural, perfusion and metabolic status of the respiratory system were assessed by *in vivo* imaging. A small animal tri-modal imaging device (Triumph, TriFoil Imaging, Chatsworth, CA, USA) was used for all imaging acquisition. After 5 h of mechanical ventilation, animals were transferred to the imaging device in supine position to acquire CT imaging of the lung. To assess the regional distribution of lung perfusion, ^99m^Tc-labeled albumin macroaggregates (mean activity 33.3±3.9 MBq/kg), were injected in the jugular vein, and SPECT imaging of the lung was performed 15 min later. Finally to assess the regional distribution of inflammatory activity (6), fluorodeoxyglucose (^18^F-FDG, mean activity 35.2±3.0 MBq/kg), was injected into the ear vein. Following a 60-min uptake period, PET imaging of the lung was acquired.

*Image analysis*

During post-processing, images obtained with CT, PET and SPECT were spatially registered and the lung was semi-automatically segmented using ITK-Snap (*itksnap.org*). Binary masks were created based on the density histogram of the CT, where the thresholds between three zones were manually marked (well aerated, poorly aerated and non-aerated). These masks were later applied to the PET and SPECT images. Lung radiodensity as well as PET and SPECT activity were calculated and compared in these three regions and also in the dorsal and ventral half of the lung using a custom-made script written in MATLAB (version R2018a, Mathworks Inc, Natick, MA, USA). PET and SPECT activity were expressed as standardized uptake value (SUV), after corrections for radioactive decay occurring after injection and before detection and for animal body weight. SUV values were normalized for the voxelwise fraction of lung tissue, as described previously (7) to more appropriately represent the metabolic activity of the lung tissue regardless of the degree of aeration (7, 8).

*Bronchoalveolar lavage*

The cell content of the bronchoalveolar lavage fluid (BALF) was analyzed as detailed previously (9). Following euthanasia and clamping of the left main bronchus, a small catheter was introduced into the right bronchus through the tracheal tube. Pre-heated (38°C) phosphate-buffered saline (PBS) with 1% bovine serum albumin (BSA) was used to wash the left lung. The injected BALF was recovered gently using gravity as fully as possible and was centrifuged at 412 G for 5 min at 5°C and the supernatant stored at –20°C until analysis. The cell pellet was re-suspended in PBS/BSA and Cytospin preparations were obtained by centrifugation at 58 *G* for 7 min. The slides were fixed, and May-Grünwald-Giemsa staining was applied for differential cell counting. Cells were counted using image acquisition software (Panoramic viewer, 3DHISTECH Ltd, Budapest, Hungary). As the distribution of the cells was not homogeneous, the cells were counted within rectangles with an edge length equivalent to the radius of the circular Cytospin. The number of cells was normalized to the surface area of the rectangles. Enzyme-linked immunosorbent assay (ELISA) analysis was performed on undiluted BALF supernatant to assess the presence of the inflammatory cytokines TNF‑α (MyBiosource MBS2021700, San Diego, CA, USA), IL‑1β, IL‑6 and IL‑8 (Raybiotech Norcross, GA, USA). Measurements were performed according to the manufacturer’s instructions.

*Lung histology*

Formaldehyde, 4%, at a hydrostatic pressure of 20 cmH_2_O was filled into the left lung. Apical, middle and basal lobe regions of the left lung were excised and fixed before embedding them in paraffin. Lung tissue sections (5 µm) were stained with hematoxylin and eosin. An expert technician who was blinded to group allocations performed the analysis in accordance with American Thoracic Society guidelines (10). Histological status of the lung was quantified using scores for the presence of neutrophils in the alveolar and interstitial spaces, the presence of hyaline membranes, proteinaceous debris filling the airspaces and alveolar septal thickening. A histology score was determined for each lung region separately (apical, middle and basal), and averaged to obtain an overall lung injury score.

**Sample size**

Since the primary outcomes included changes in respiratory tissue elastance (H), the sample size was estimated based on our previous data on this parameter (9) to detect 20% between-group differences; assuming a coefficient of variation of 15% in the injured lung and 8% in the healthy lungs, a statistical power of 0.8 and 2-sided alpha error of 0.05. The estimation resulted in a required sample size of 9 rabbits for the injured groups. Considering the smaller variability in the healthy rabbits, a sample size of 6 was calculated for these groups. To take into account potential drop-outs, we included 7 animals in each healthy group and 12 animals in each injured group.

**Allocating animals to experimental groups**

A block randomization procedure was used to assign the animals to one of the four experimental groups. The website randomizer.org was used to generate the blocks.

**Statistical methods**

Data are presented as mean ± half-width of 95% confidence interval. Normality of the data was assessed for each variable with the Shapiro-Wilk test. In case of a failed normality test, the variable was log-transformed. Three-way repeated measures analyses of variance (ANOVA) using linear mixed-effect model fits by a restricted maximum likelihood (REML) method were applied to analyze respiratory mechanical, blood gas, and cytokine data with factors ventilation mode (PCV or PVV), injury (healthy or ARDS) and time. In case of a significant test, Dunnett’s post-hoc tests were used for time (using T0 as a reference level), ventilation mode (using PCV as reference) and injury (using control as reference). Imaging parameters were analyzed using three-way repeated measures ANOVA with factors ventilation mode, injury and aeration zone or position (dorsal or ventral). Histology lung injury score, global CT density, cytokine levels and BALF cell counts were analyzed using two-way ANOVA with Holm-Sidak post-hoc tests using injury and ventilation mode as between-group variables. The statistical tests were performed within the R environment with the *lme4*, *lsmeans* and *stats* packages and SigmaPlot (version 13, Systat Software, Inc. Chicago, IL, USA). The statistical tests were performed with a significance level of p < 0.05, and all p values were two-sided.

RESULTS

*Respiratory mechanics*

Parameters characterizing respiratory mechanics obtained prior to initiating the 5-hour ventilation are displayed in Table S1. No significant differences were observed in any parameter between the study groups under the BL conditions. Induction of lung injury led to significant increases in tissue damping and elastance in groups PCV-ARDS and PVV-ARDS (p<0.001) with no difference between the two ARDS groups.

*Morphological findings*

Lung injury scores, summarized in Table S2, were significantly elevated in animals with ARDS (0.79±0.01, 0.78±0.01 for the PCV-ARDS and PVV-ARDS groups, respectively) compared to those with healthy lungs (0.43±0.02 for both control groups, p<0.05). Differences in lung injury scores were also observed for the global CT density, which reflects the amount of well aerated zones, with values being significantly higher in the ARDS groups (375±32 mean pixel value (MPV) and 382±31 MPV for PCV-ARDS and PVV-ARDS groups, respectively) than those obtained in healthy controls (306±51 MPV and 298±23MPV for PCV-CTRL and PVV-CTRL groups, respectively, p<0.05).

*Molecular and cellular content of bronchoalveolar lavage fluid*

The results of the cellular and molecular evaluation of lung injury are summarized in Table S3. Differences in the cytokine and cell content of BALF were observed between ARDS and CTRL groups. No differences based on ventilation mode were detected.

*Ancillary analysis*

Ventilation parameters characterizing the 5-hour experimental period (VT, RR) are depicted in Figures S1-S3. Significantly lower values of inspiratory driving pressure (IDP) were required to maintain the same minute ventilation in healthy animals (p < 0.01, ARDS vs. CTRL), with a possibility of progressive reduction in IDP in the PVV-CTRL group (p < 0.01 vs. T0). Such reduction was not observed in the PCV-CTRL group. No differences were detected in the hemodynamic parameters (MAP, CVP, HR) between the ventilation modes during the 5-hour ventilation period (Figure S4).

**Figure S1**

Trace of the physiological breathing pattern applied in groups PVV-ARDS and PVV-CTRL during the 5-hour ventilation period. AU: arbitrary unit.


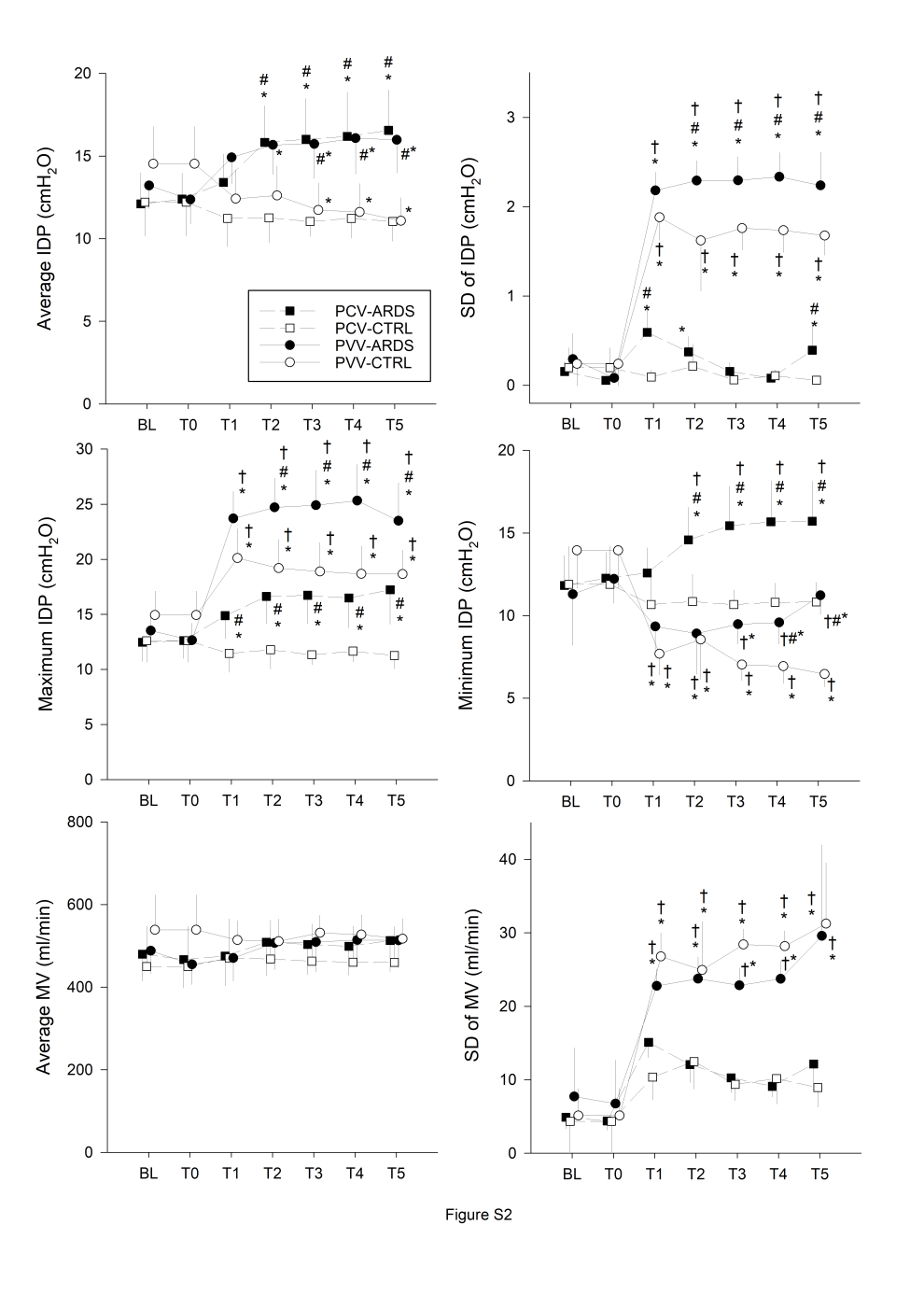


**Figure S2**

Ventilation parameters obtained before and during the 5-hour long ventilation period. Values expressed as mean ± half-width of 95% confidence interval.

IDP: inspiratory driving pressure, MV: minute ventilation, SD: standard deviation.

BL: baseline, T0: immediately after induction of lung injury, 1H-5H: average value during the corresponding hour of the 5-hour long ventilation period.

PCV: pressure-controlled ventilation, PVV: physiological variable ventilation, ARDS: presence of lung injury, CTRL: absence of lung injury.

*: p < 0.05 vs. T0, #: p < 0.05 vs. CTRL, †: p < 0.05 vs. PCV.


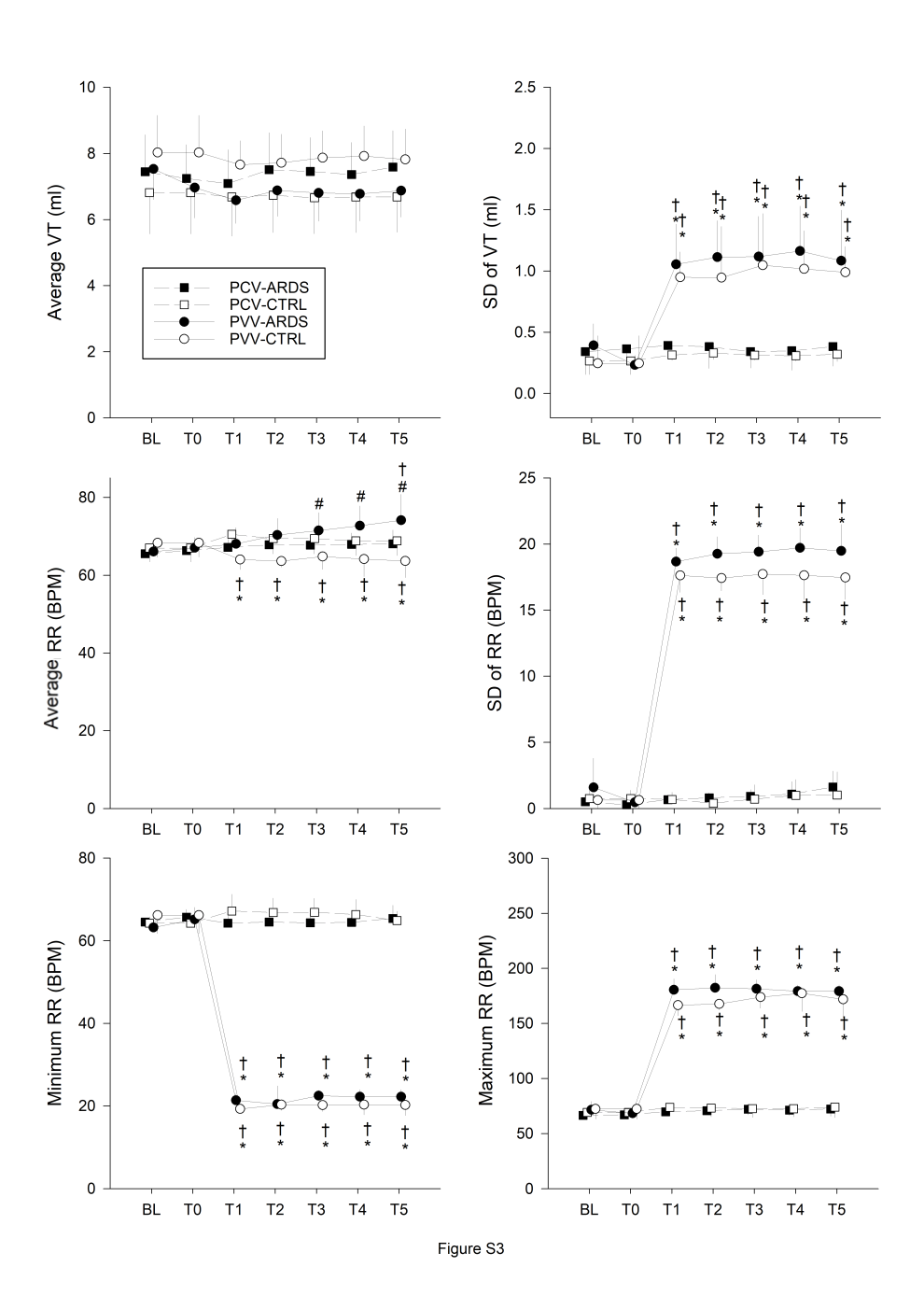


**Figure S3**

Ventilation parameters obtained before and during the 5-hour long ventilation period. Values expressed as mean ± half-width of 95% confidence interval.

VT: tidal volume, RR: respiratory rate, BPM: breath per minute, SD: standard deviation.

BL: baseline, T0: immediately after induction of lung injury, 1H-5H: average value during the corresponding hour of the 5-hour long ventilation period.

PCV: pressure-controlled ventilation, PVV: physiological variable ventilation, ARDS: presence of lung injury, CTRL: absence of lung injury.

*: p < 0.05 vs. T0, #: p < 0.05 vs. CTRL, †: p < 0.05 vs. PCV, n.s.: not significant.

**Figure S4**

Haemodynamic parameters obtained before and during the 5-hour long ventilation period. Values expressed as mean ± half-width of 95% confidence interval.

MAP: mean arterial pressure, CVP: central venous pressure, HR: heart rate, BPM: beat per minute.

BL: baseline, T0: immediately after induction of lung injury, 1H-5H: average value during the corresponding hour of the 5-hour long ventilation period.

PCV: pressure-controlled ventilation, PVV: physiological variable ventilation, ARDS: presence of lung injury, CTRL: absence of lung injury.

*: p < 0.05 vs. T0, #: p < 0.05 vs. CTRL, †: p < 0.05 vs. PCV, n.s.: not significant.

|  | PCV-CTRL | | PVV-CTRL | | PCV-ARDS | | PVV-ARDS | |
| --- | --- | --- | --- | --- | --- | --- | --- | --- |
|  | BL | T0 | BL | T0 | BL | T0 | BL | T0 |
| Raw (cmH_2_O.s/l) | 36.5±9.7 | 36.5±9.7 | 34.2±6.4 | 34.2±6.4 | 37.2±4.4 | 34.1±5.4 | 38.5±3.5 | 36.8±3.7 |
| G (cmH_2_O/l) | 315±81 | 315±81 | 245±35 | 245±35 | 293±49 | 336±52 * | 291±33 | 338±33 *# |
| H (cmH_2_O/l) | 1028±174 | 1028±174 | 894±151 | 894±151 | 955±104 | 1291±181*# | 958±122 | 1353±75 *# |

**Table S1**

Values of respiratory mechanical parameters at baseline (BL) and immediately after induction of lung injury (T0). Values expressed as mean ± half-width of 95% confidence interval.

Raw: airway resistance, G: respiratory tissue damping, H: respiratory tissue elastance.

PCV: pressure-controlled ventilation, PVV: physiological variable ventilation, ARDS: presence of lung injury, CTRL: absence of lung injury.

*: p < 0.05 vs. BL, #: p < 0.05 vs. CTRL

|  | PCV-CTRL | PVV-CTRL | PCV-ARDS | PVV-ARDS |
| --- | --- | --- | --- | --- |
| Global CT density (AU) | 306±51 | 298±23 | 375±32 # | 382±31 # |
| Lung injury score | 0.43±0.02 | 0.43±0.02 | 0.79±0.01 # | 0.78±0.01 # |

**Table S2**

Morphological indices of lung injury. Values expressed as mean ± half-width of 95% confidence interval. AU: arbitrary unit.

PCV: pressure-controlled ventilation, PVV: physiological variable ventilation, ARDS: presence of lung injury, CTRL: absence of lung injury.

#: p < 0.05 vs. CTRL

|  | PCV-CTRL | PVV-CTRL | PCV-ARDS | PVV-ARDS |
| --- | --- | --- | --- | --- |
| IL-1β (pg/ml) | 116.5±32.1 | 126.2±37.8 | 168.5±7.6 # | 170.7±17.5 # |
| IL-6 (pg/ml) | 3.17±1.89 | 4.93±5.77 | 8.67±2.89 # | 9.93±2.77 # |
| IL-8 (pg/ml) | 113.0±49.3 | 164.8±79.8 | 245.5±11.6 # | 242.8±19.3 # |
| TNF-a (pg/ml) | 15.01±3.29 | 23.18±24.86 | 18.38±1.35 | 22.99±10.51 |
| Protein (mg/ml) | 0.13±0.17 | 0.49±1.09 | 0.78±0.25 # | 1.00±0.44 # |
|  |  |  |  |  |
| Macrophages (%) | 79.1±11.4 | 67.5±7.1 | 40.5±12.5 # | 34.0±10.4 # |
| Neutrophils (%) | 12.4±7.4 | 19.5±9.1 | 51.9±14.3 # | 60.1±11.0 # |
| Lymphocytes (%) | 8.3±5.3 | 12.4±4.0 | 7.3±3.2 | 5.5±1.3 # |

**Table S3**

Cytokine and cell content of the bronchoalveolar lavage fluid. Values expressed as mean ± half-width of 95% confidence interval.

PCV: pressure-controlled ventilation, PVV: physiological variable ventilation, ARDS: presence of lung injury, CTRL: absence of lung injury.

#: p < 0.05 vs. CTRL.

REFERENCES - Online data supplement

1. Ards Definition Task Force, Ranieri VM, Rubenfeld GD, et al. Acute respiratory distress syndrome: the Berlin Definition. JAMA 2012;307(23):2526-2533.

2. Fodor GH, Bayat S, Albu G, et al. Variable Ventilation Is Equally Effective as Conventional Pressure Control Ventilation for Optimizing Lung Function in a Rabbit Model of ARDS. Front Physiol 2019;10:803.

3. Franken H, Clement J, Cauberghs M, et al. Oscillating flow of a viscous compressible fluid through a rigid tube: a theoretical model. IEEE Trans Biomed Eng 1981;28(5):416-420.

4. Hantos Z, Daroczy B, Suki B, et al. Input impedance and peripheral inhomogeneity of dog lungs. J Appl Physiol (1985) 1992;72(1):168-178.

5. Petak F, Hantos Z, Adamicza A, et al. Methacholine-induced bronchoconstriction in rats: effects of intravenous vs. aerosol delivery. J Appl Physiol (1985) 1997;82(5):1479-1487.

6. Capitanio S, Nordin AJ, Noraini AR, et al. PET/CT in nononcological lung diseases: current applications and future perspectives. European respiratory review : an official journal of the European Respiratory Society 2016;25(141):247-258.

7. Holman BF, Cuplov V, Millner L, et al. Improved correction for the tissue fraction effect in lung PET/CT imaging. Phys Med Biol 2015;60(18):7387-7402.

8. Lambrou T, Groves AM, Erlandsson K, et al. The importance of correction for tissue fraction effects in lung PET: preliminary findings. European journal of nuclear medicine and molecular imaging 2011;38(12):2238-2246.

9. Walesa M, Bayat S, Albu G, et al. Comparison between neurally-assisted, controlled, and physiologically variable ventilation in healthy rabbits. Br J Anaesth 2018;121(4):918-927.

10. Matute-Bello G, Downey G, Moore BB, et al. An official American Thoracic Society workshop report: features and measurements of experimental acute lung injury in animals. American journal of respiratory cell and molecular biology 2011;44(5):725-738.
